# IMPACT OF A HEARING INTERVENTION VERSUS HEALTH EDUCATION CONTROL ON FATIGUE SYMPTOMS OVER THREE YEARS

**DOI:** 10.1093/geroni/igae098.0612

**Published:** 2024-12-31

**Authors:** Sarah Bessen

**Affiliations:** Johns Hopkins University, Baltimore, Maryland, United States

## Abstract

Fatigue is a common complaint among older adults with hearing loss. We investigated the effect of hearing intervention versus health education control on fatigue over 3 years in older adults with hearing loss using the Aging and Cognitive Health Evaluation in Elders (ACHIEVE) study. A total of 977 adults (mean age: 76.8 years, range 70-84; female: 53.5%; White: 87.8%) with hearing loss were recruited from the Atherosclerosis Risk in Communities Study (ARIC) (n=238) and de novo from the community (n=739). Fatigue symptoms were measured with the PROMIS fatigue bank and RAND-36 questionnaires at baseline, 1-year, 2-year, and 3-year follow-up visits (for PROMIS-score, higher=more fatigue, whereas for RAND-score, higher=less fatigue). The intervention effect was estimated as the differences in the 3-year change in fatigue scores between groups using linear mixed-effects models. Over 3 years, preliminary results suggest there was potential beneficial effect of the hearing intervention vs. health education control on change in the PROMIS-fatigue score (β=-0.32 [95% CI: -1.17, 0.50]) and the RAND fatigue score (β=0.16 [95% CI: 0.16 0.04, 0.29]). Stratification by recruitment source showed consistent results with a stronger magnitude of the effect among de novo compared to ARIC participants. Overall, our findings suggest a possible beneficial effect of the hearing intervention on fatigue symptoms over 3 years. This research adds to current evidence supporting the wide-ranging benefits of hearing care and informs policy efforts to increase awareness and accessibility.

